# Translating acceptability to sustained delivery: Clinician and manager perspectives on implementing modified constraint‐induced movement therapy in an early‐supported discharge rehabilitation service

**DOI:** 10.1111/1440-1630.12993

**Published:** 2024-10-07

**Authors:** Ashan Weerakkody, Erin Godecke, Barby Singer

**Affiliations:** ^1^ Department of Health Rehabilitation in the Home, South Metropolitan Health Service Fremantle Western Australia Australia; ^2^ School of Medical and Health Sciences Edith Cowan University Joondalup Western Australia Australia; ^3^ Department of Health Sir Charles Gairdner Hospital, North Metropolitan Health Service Nedlands Western Australia Australia

**Keywords:** implementation science, occupational therapy, physiotherapy, qualitative research, stroke rehabilitation, upper extremity

## Abstract

**Background:**

Modified constraint‐induced movement therapy (mCIMT) improves upper limb (UL) function after stroke. Despite up to one‐third of stroke survivors being eligible, clinical uptake remains poor. To address this, a multi‐modal behaviour change intervention was implemented across a large seven‐site early‐supported discharge (ESD) rehabilitation service. This study investigated the acceptability of mCIMT implementation within this ESD service and identified adaptations required for sustained delivery.

**Methods:**

This qualitative study was nested within a mixed‐methods process evaluation of mCIMT implementation. Four focus groups (*n* = 24) comprising therapists (two groups), therapy assistants (one group), and allied health managers (one group) were conducted. Data were analysed using reflexive thematic analysis and mapped to the Theoretical Domains Framework (TDF).

**Consumer and Community Involvement:**

Consumers were not directly involved in this study; however, lived experience research partners have helped shape the larger mixed‐methods implementation study.

**Findings:**

Four themes were generated and mapped to the TDF. Factors related to acceptability included interdisciplinary practice in sharing workloads (belief about capabilities), practice opportunities across a range of UL presentations (skills), clinician attitudes influencing patient engagement (optimism), time constraints (belief about consequences), and cognitive overload from multiple systems and processes (memory, attention, and decision‐making processes). Factors facilitating sustained delivery included improving stroke survivor education (knowledge), sharing success stories across teams (reinforcement), manager facilitation (social/professional role and identity), and the perception that the ESD setting was optimal for mCIMT delivery (social influences).

**Conclusion:**

mCIMT was acceptable in the ESD service, with clinicians feeling a responsibility to provide it. Key adaptations for sustained delivery included ongoing training, resource adaptation, and enhanced patient and carer engagement. Successful implementation and sustained delivery of mCIMT in the ESD service could enhance UL function and reduce the burden of care for potentially hundreds of stroke survivors and their carers.

**PLAIN LANGUAGE SUMMARY:**

Modified constraint‐induced movement therapy (mCIMT) helps improve arm movement after a stroke. However, many stroke survivors do not get this therapy. To fix this, we started a program in a large home‐based rehabilitation service. This study looked at how well mCIMT could fit into this service. We also wanted to know what changes were needed to make sure it was regularly provided.

We held four group discussions with therapists, therapy assistants, and health managers. A total of 24 people took part.

From these discussions, we found several important points. Therapists needed to work together as a team. They also needed to practice mCIMT to get better at delivering it. Therapists having a positive attitude would encourage more stroke survivors to take part. For long‐term success, stroke survivors need better education about mCIMT. Managers need to encourage therapists to provide mCIMT. The rehabilitation service should also share their success stories about this therapy to encourage therapists to deliver it and stroke survivors to ask for it.

Therapists enjoyed delivering mCIMT in the rehabilitation service. It worked better than other therapies to improve a stroke survivor's arm function. Because of this, they also felt it was their duty to offer mCIMT. Having ongoing training and better resources would help keep mCIMT going. If mCIMT can be provided regularly in this service, it could lead to better arm function and less care needed for many stroke survivors and their carers.

**Key Points for Occupational Therapy**

- Challenges persist in delivering mCIMT despite strong evidence for improving arm function in stroke survivors.
- mCIMT was acceptable for routine delivery within an ESD service.
- Sustained delivery of mCIMT requires engagement from all stakeholders, ongoing training, clinical support, and management facilitation.

## 1 INTRODUCTION

Modified constraint‐induced movement therapy (mCIMT) is an effective rehabilitation intervention to improve upper limb (UL) function after stroke (Kwakkel et al., 2015), with up to one‐third of stroke survivors eligible to participate (Christie, Rendell, Fearn, et al., 2023). Although mCIMT is supported by strong evidence and recommendations in several national stroke clinical practice guidelines (Intercollegiate Stroke Working Party, 2023; Stroke Foundation, 2024; Teasell et al., 2020), its translation into routine clinical practice remains challenging (Weerakkody, White, et al., 2023). Several barriers hinder the provision of mCIMT in rehabilitation services internationally. These include therapist factors such as the lack of knowledge and skills to confidently deliver mCIMT and institutional factors such as insufficient staffing, resources, and management support (Christie, Rendell, McCluskey, et al., 2023; Weerakkody, White, et al., 2023). Rehabilitation therapists are known to be the gatekeepers in determining who does or does not receive mCIMT. Most studies have focussed on therapist populations with limited experience with CIMT or its modified versions, leaving a gap in understanding the perspectives of more experienced therapists (Christie, Rendell, McCluskey, et al., 2023; Weerakkody, White, et al., 2023). This limited familiarity with mCIMT may obscure other potential factors influencing its acceptability, beyond the lack of knowledge and skills previously identified as primary barriers.

Building on the existing understanding of barriers, our research group developed and delivered a behaviour change intervention within Rehabilitation in the Home (RITH), a large, multi‐site early‐supported discharge (ESD) rehabilitation service in Perth, Australia. This intervention aimed to sustainably increase the delivery of mCIMT to eligible stroke survivors within existing funding and staffing levels, that is, without additional resources dedicated to this project (Weerakkody, Emmanuel, et al., 2023). As part of this intervention, therapists completed a survey to identify barriers and enablers to routine mCIMT provision within the service. The survey findings echoed previous literature, highlighting therapists' lack of skills and confidence, time and staffing constraints, and the challenge of prioritising UL rehabilitation alongside other rehabilitation goals, such as improving mobility or cognitive function. Barriers and enablers were mapped to the Theoretical Domains Framework (TDF) (Atkins et al., 2017), which guided the selection of behaviour change techniques using the Behaviour Change Wheel (BCW) (Michie et al., 2014). After developing a context‐specific mCIMT protocol, a multi‐modal intervention was delivered to therapists, including education, training, modelling of best practice, and resource development. This staged implementation has been published previously (Weerakkody, Emmanuel, et al., 2023).

Implementation of evidence‐based rehabilitation interventions in clinical settings is an iterative process (Morris et al., 2019). The training package, resource development, and adaptations to the original CIMT protocol were developed using data collected from clinicians with minimal mCIMT experience (Weerakkody, Emmanuel, et al., 2023). As therapists gain knowledge and experience in delivering mCIMT programs, additional factors may emerge that influence its acceptability, providing feedback to guide further program improvements. This knowledge can be leveraged to sustain delivery and embed mCIMT into routine clinical practice in the service.

This study aimed to explore the in‐depth perceptions and experiences of RITH therapists, therapy assistants, and allied health managers regarding the acceptability of mCIMT implementation within their service. Specifically, this study sought to identify factors facilitating or hindering the routine delivery of mCIMT and gain insights for further program adaptations that may be required to support sustained mCIMT delivery in this service.

## 2 METHODS

### 2.1 Design

This qualitative descriptive study was nested within a mixed‐methods process evaluation of mCIMT implementation within an ESD service in Western Australia. This study was registered on the Australian New Zealand Clinical Trials Registry (ACTRN12620000079943), and the consolidated criteria for reporting qualitative research (COREQ) guidelines were used in the design and reporting of this study (Tong et al., 2007). Ethical approval was obtained from the South Metropolitan Health Service (RGS0000003058) and Edith Cowan University Human Research Ethics Committees (2019‐00989). All participants gave informed consent prior to participating in this study.

### 2.2 Setting and mCIMT implementation

The ESD service, RITH, operated across seven bases in a metropolitan region. Approximately 700 stroke survivors are admitted to the RITH service annually. The mCIMT protocol was a minimum of 2 weeks in duration, with three 1‐hour sessions per week with a therapist and/or therapy assistant. As part of the behaviour change intervention, therapists (physiotherapists and occupational therapists) and therapy assistants attended four 90‐minute practical workshops, conducted between October 2018 and November 2019. They also received expert peer modelling with a P3 neurological clinician (see below for definition) for at least one full mCIMT program. Ongoing clinical support and additional peer modelling were provided to therapists as needed. Further details about the service, the RITH mCIMT protocol, and the behaviour change intervention delivered to clinicians have been reported elsewhere (Weerakkody, Emmanuel, et al., 2023).

### 2.3 P3 neurological physiotherapist and occupational therapist

The P3 designation is an advanced level of allied health practice in Western Australia Health, signifying post‐graduate qualifications and extensive experience in a clinical area. During the time of this study, there was one P3 neurological physiotherapist working full‐time and one P3 neurological occupational therapist working 3 days per week, supporting approximately 80 clinicians across all RITH bases. Their role involved providing clinical support to RITH physiotherapists and/or occupational therapists, leading service improvement initiatives, and delivering continuing education relevant to neurological rehabilitation. Developing and delivering the mCIMT behaviour change intervention was undertaken by the P3 clinicians in addition to their usual workload.

### 2.4 Sample

Purposive sampling was used to recruit RITH therapists (physiotherapists and occupational therapists) and therapy assistants, who were eligible if they had attended the RITH mCIMT training program and/or had been involved in delivering at least one mCIMT program with a stroke survivor. Convenience sampling was used to recruit allied health managers. Managers were eligible if they were either an area manager or site‐based coordinator acting as line managers to RITH therapy staff. A. W. contacted potential participants via email, including a letter of invitation to join the study. Those who expressed interest received a participant information and consent form to review before consenting. Participants were informed that their participation was entirely voluntary and that their decision would not affect their current or future employment. Participants were recruited from all seven RITH sites to ensure that views expressed were representative of the entire service.

### 2.5 Data collection

Focus groups were conducted using a semi‐structured guide developed by the research team (Supporting Information 1, 2, and 3). Separate focus groups were conducted for rehabilitation therapists, managers, and therapy assistants, to reflect the varying influence each cohort has on implementing mCIMT. Questions were developed and mapped to the domains and constructs of the TDF to ensure the question guide provided opportunities to cover all domains. Focus groups were audio‐recorded and transcribed verbatim, and participants were offered the opportunity to review their transcripts for accuracy (member checking).

E. G. (PhD, speech pathologist, female) facilitated three focus groups (two with therapists and one with managers), and B. S. (PhD, physiotherapist, female) facilitated the focus group with therapy assistants. Only the focus group participants and the facilitator were present during each focus group. E. G. and B. S. are experienced stroke rehabilitation clinicians and researchers. Both facilitators had no direct involvement with the study participants.

### 2.6 Data analysis

The interview transcripts were imported into NVivo 12 (QSR International Pty Ltd, 2018) for analysis. Reflexive thematic analysis was conducted by A. W. (PhD candidate, physiotherapist, male) and an independent research assistant (BSc, no health background, female), following Braun and Clarke's approach (Braun & Clarke, 2022). Reflexive thematic analysis allows for a rich, detailed, and complex account of the data, emphasising the active role of the researcher in generating themes from the data (Braun & Clarke, 2019). This approach was particularly suitable for this exploratory study where the researcher's interpretation was integral in understanding the experiences of mCIMT among participants. An inductive approach was used to develop codes and themes from the data, progressing through the six defined phases of reflexive thematic analysis iteratively (Braun & Clarke, 2022). A. W. and the research assistant independently familiarised themselves with the data and coded all focus groups. They then met to discuss additional codes not generated by the other person. Similar codes were grouped to generate initial themes by A. W., which were further refined through coding and discussion with the research team to develop and finalise themes.

Themes were then mapped to the TDF. The TDF allows for the systematic exploration of 14 key domains, including knowledge, skills, social influences, and environmental context and resources, among others, which encompass a wide range of psychological and organisational factors that can influence behaviour in rehabilitation settings (Cane et al., 2012; French et al., 2012). This framework was chosen to identify any further factors influencing the acceptability of mCIMT and to facilitate the design of effective strategies to sustain the delivery of mCIMT in the RITH service (Atkins et al., 2017). These factors were then categorised as either being related to reflections on the implementation of mCIMT (exploring acceptability) or to improving the mCIMT program delivery (supporting sustainability). Mapping to the TDF was conducted by A. W. in collaboration with the research team.

### 2.7 Consumer and community involvement

The implementation of mCIMT in this service has actively engaged all relevant stakeholders during each phase, from the initial design and data analysis to the ongoing refinement of program delivery. Clinician involvement was integral in the development and delivery of the behaviour change intervention (Weerakkody, Emmanuel, et al., 2023), for which this study investigates their experiences for further insights and enhancements. A concurrent study was undertaken with stroke survivors and carers to understand their experiences related to the acceptability of mCIMT in the ESD setting (Weerakkody et al., 2024). That study involved three lived experience partners throughout its design and analysis of findings.

### 2.8 Reflexivity and trustworthiness

Reflexivity is integral to thematic analysis, requiring the researcher to acknowledge and address their personal biases, preconceptions, and experiences to enhance the accuracy and reliability of the findings (Braun & Clarke, 2019, 2022, 2023). A. W., a neurological physiotherapist who co‐developed and delivered the behaviour change intervention to RITH clinicians, maintained a reflexive journal to facilitate this process. In the journal, he recorded his thoughts, feelings, and decision‐making processes during the generation of themes, and he reflected on their potential impact on the analysis (Braun & Clarke, 2022).

Several other strategies were employed to maximise the methodological rigour and trustworthiness of the findings (Nowell et al., 2017; Smith & McGannon, 2017). Both the data (transcripts) and findings (themes) were checked by participants to enhance credibility. A research assistant, independent to the study, operated as a second coder. This measure was taken to mitigate any potential overshadowing of participant experiences by A. W. resulting from his role in developing the behaviour change intervention (Noble & Smith, 2015). This also brought a unique perspective to the analysis, facilitating the identification of additional codes and ensuring a more thorough analysis of the data. Further, the research team engaged in discussions at every phase of the study, from the design and development of interview guides to inductive coding and deductive mapping. An audit trail was maintained to document these processes.

### 2.9 Positionality statement

In addition to describing the reflexive processes undertaken by A. W. in his role working for the RITH service as the P3 neurological physiotherapist, it is pertinent to detail the positionality of all authors. B. S. is an experienced neurological physiotherapist who has worked in academia for the past 20 years. She has considerable experience in the delivery of evidence‐based physical rehabilitation interventions in both her prior clinical and current academic roles. E. G. is a speech pathologist who works in clinical and academic settings. She possesses extensive experience in the application and evaluation of implementation science frameworks within multi‐disciplinary allied health practice. Both B. S. and E. G. have coordinated post‐graduate neurological rehabilitation courses and supervised higher degree research students in the implementation of evidence‐based practice in stroke. Neither B. S. nor E. G. have worked for the RITH service. The experience of both B. S. and E. G. was complementary in understanding evidence‐based practice, and the theory of behaviour involved in implementation, which combined with A. W.'s knowledge of the service, was essential in understanding the acceptability and sustainability of mCIMT in the RITH service.

## 3 RESULTS

All four focus groups were conducted during working hours in seminar rooms of metropolitan teaching hospitals between August 2020 and February 2021. Initially, these focus groups were scheduled to take place shortly after the delivery of the behaviour change intervention in early 2020. However, because of the COVID‐19 pandemic, they were delayed by approximately 6 months. Focus group durations ranged from 41 to 64 minutes.

### 3.1 Participants

Sixteen rehabilitation therapists were invited to participate in the study, with 13 participating in one of two therapist focus groups (seven physiotherapists/six occupational therapists; 12 female/one male). All RITH sites were represented by at least one therapist. One physiotherapist declined to participate, one occupational therapist was unable to attend because of a conflict with another appointment, and one occupational therapist who had agreed to participate was unwell on the day of the focus group and unable to participate. Therapists ranged in post‐graduate clinical experience between 6 and >20 years, with time in the RITH service ranging between 2 and >10 years.

The managers' focus group contained one area manager and six site‐based coordinators (*n* = 7, all female). One area manager and one site‐based coordinator did not participate because of other commitments. The clinical discipline backgrounds of the managers' group included four physiotherapists, one occupational therapist, one speech pathologist, and one dietitian. Four allied health assistants were invited, and all consented to participate in the therapy assistant focus group (*n* = 4, three female/one male). The time working in the RITH service for therapy assistants ranged between 1 and 6 years.

### 3.2 Themes

Reflexive thematic analysis led to the generation of four themes: (1) clinical practice and knowledge application; (2) patient challenges and engagement; (3) service delivery constraints and suggestions to overcome; and (4) program implementation and integration into routine care.

Table 1 demonstrates the determinants of implementation for mCIMT, reflecting its acceptability in the RITH service, mapped to the TDF. Table 2 maps the determinants supporting sustained delivery of mCIMT, also to the TDF. These tables highlight factors that were most frequently discussed in focus groups and areas of consensus among participants. Each determinant is accompanied by illustrative quotes to provide insights into participant perspectives and experiences.

**TABLE 1:** Factors related to acceptability of mCIMT provision.

Theoretical domain	Factors associated with acceptability to mCIMT provision	Example quote and associated TDF construct
Theme 1: Clinical practice and knowledge application		
Knowledge	Clear understanding about the individual components that make up the mCIMT package	*Prior to the implementation of the RITH mCIMT [program] I thought it was much more about the actual constraint‐related practice, whereas what we've learnt … is that it's really about the behavioural contract and the educational content and getting the ‘buy in’ with the patient and making them understand how much they're using their limb, and with my experience so far, it seems to be the thing that has the biggest effect on the patient's outcome*. (PT1, procedural knowledge)
Skills	Practice opportunities are required to develop mCIMT‐specific skills for a variety of UL presentations	*You've sort of got to see a mild upper limb, maybe a more moderately affected upper limb … So I think once you've sort of covered those bases you might have a bit more of a toolkit in how to approach different scenarios*. (PT7, skills development)
Social/professional role and identity	Belief that clinical practice should follow clinical practice guideline recommendations	I do think it is the responsibility of management and coordinators. This is the best practice, somebody needs to be doing it in WA for these people *laughs* ‐ so I do think there is a role to do it [role of managers], and I know it's not easy and it's time‐intensive but I think for the gains that we have, it's important. (MC1, leadership)
Beliefs about capabilities	Interdisciplinary collaboration shares workload and accommodates part‐time staff	*As a part‐time staff, like three days a week is perfect because I can just do the one day and then I get TA or Physio to the other two days … once it's all set up you just go in and smash it out, you do all the things you set up and then you leave, like it's all done … it's like actually even easier than having a regular rehab session*. (OT1, self‐efficacy)
Beliefs about consequences	Perceived time constraints persist based on clinician attitude to mCIMT	*If you don't believe [in mCIMT], like, if you believe it’s too time consuming and it's really difficult with your time management skills, then you're not going to sell it, are you, because it's not really going to succeed*. (PT2, consequents)
Optimism	The behaviour change associated with mCIMT leads to superior outcomes	*I explain it to the patients like it's the gold standard of your upper limb therapy, everything else comes below that. This is the behavioural change that will make a real difference to your arm if you stick to it*. (OT2, optimism)
Goals	Difficulty balancing other rehabilitation goals such as mobility with mCIMT provision	*… sometimes for physios if you've got a patient that's really impaired and they've got mobility issues, lower limb, … tone, walking stuff, balance, cerebellar, vestibular, there's a lot going on, it is hard to know at what point you introduce CIMT, and what you prioritise …* (PT7, goal/target setting)
Memory, attention, and decision processes	Clinicians should have autonomy to offer mCIMT	*I think you have the clinical decision as to whether they're suitable or not, so I don't think they should make it mandatory. [mCIMT for eligible patients]* (PT3, decision making)
Theme 2: Patient challenges and engagement		
Knowledge	Lack of patient awareness of mCIMT	*I'm surprised about the amount of patients who come out of hospital and have never heard of it*. (OT2, knowledge)
Environmental context and resources	Carer support is important to facilitate sufficient practice	*So I think you have to get the whole family on side really or whoever they're living with, to encourage that [practice intensity], and that's where I have heard the pitfalls are is if they just don't have that support, they're not going to get enough from us just doing it with them*. (MC3, resources)
Goals	Importance of setting meaningful goals to enhance engagement	*What did they use the hand for before, obviously if they had a job or if they were a stay‐at‐home parent then you could focus on that goal as well and what they want to work on …* (OT1, goals autonomous/controlled)
Emotions	Affect can change during the program	*We had one lady who just found it so fatiguing by the end of every day and it did get easier across the two weeks, but, she would just say to us ‘gosh this is so much and my brain is working so hard all the time, I can really feel the fatigue afterwards’ but then she did really well and she was really happy afterwards, so that was good*. (PT1, positive/negative affect)
Beliefs about consequences	Development of shoulder pain	*Pain has been a big issue with a lot of patients, who've developed shoulder issues, whether they've been underlying there before or not, it's hard to say … and then they've gone to the doctors and they've had scans or whatever and it's been bursitis or a tear and you do wonder whether that's because of what you've done, the repetition*. (OT6, anticipated regret)
Optimism	Clinician attitude to mCIMT can support or negate patient engagement	*I think in order for the program to work well, like, you need to really believe in it yourself … so I think that's really important ‐ your attitude to it, if you really believe in it and if you know what you're doing … it can really make a huge difference to them, whether they say yes or no, whether they succeed in getting past barriers and emotionally getting through things*. (OT1, optimism)
Theme 3: Service delivery constraints and suggested improvements		
Environmental context and resources	Time and staffing constraints	*We have had issues with appropriate levels of staffing to be able deliver the program appropriately, and that's been one of the criteria of implementing the program is that you have to be able to have the amount of staff available, the service events available, and that's definitely been an issue for us at our particular site*. (MC6, environmental stressors)
	Stroke survivor is suitable for discharge to outpatient service so misses out on mCIMT	*We don't continue treatment if they're appropriate for outpatient treatment, whether or not it's [mCIMT] available or not*. (MC5, organisational culture/climate)
Skills	Difficulty for part‐time staff to attend training sessions	*I think one of the other challenges we had is the three modules were offered and it was really difficult to capture the part time staff within that, so um, you know they tried to pick fairly consistent days so staff could go through each of the three modules but it was a bit tricky, capturing the part time staff* (MC2, skills development)
Social influences	Inequity in access to P3 neurological therapist for clinical support	*I think the P3 has an impact on it, and I think particularly when you don't have a P3 in your site or your area then trying to coordinate, particularly with part time staff, their capacity to be able to do a joint visit and support them that way is much more difficult* (M7, social support)
Intentions	Caseload constraints influence intention to provide mCIMT	*sometimes if the patient barriers are there, I feel like the therapist hasn't done a great deal to overcome the patient factors because they don't really have the capacity themselves* (MC6, stability of intentions)
Emotions	Lack of clinical psychology to support stroke survivors with low mood, frustration, and other challenges associated with the mCIMT program	*when they get frustrated and when their mood changes because they're … not able to use their hand and it's really highlighting a lot of their deficits … that might be something that would be worthwhile looking into in the future, like whether or not we can get psychology support … if we could address that … I think our program would go a lot more smoothly* (OT2, stress)
Memory, attention, and decision processes	Therapist cognitive overload and fatigue from multiple systems and service processes	*I think because we accept referrals that are everything, we have general rehab, orthopaedics, whatever ‐ the staff have got so many systems and processes, it's actually very hard to remember to do something. So you kind of do have to make it common sense or a clear role, otherwise it will fall off*. (MC1, memory)
Theme 4: Program implementation and integration into routine care		
Knowledge	The home environment provides rich opportunities to deliver mCIMT	*I might be a bit biased, but I think that we are in the best position out of all the services to do it, we've got the context rich environment, they're often coming to us as subacute [period after stroke], they're coming to terms to adapting to functional life at home and … I just feel like we're in a really good position to implement that*. (PT5, knowledge of task environment)
Belief about capabilities	Standardisation of practice area increases consistency between clinicians and saves set‐up time	*I've found also that the pack that you take out, so that it stays at the patient's house, … it's got everything together and usually patients are able to keep that with the mat and the other resources that we're using and that's made it much easier since that's all been organised too … you can walk in after a therapist has been there and understand what their homework tasks were, the sorts of things they're supposed to be achieving* (TA1, self‐efficacy)
Environmental context and resources	The service supports provision of mCIMT despite potential increases in length of stay	*RITH is really good as long as you're clinically reasoning why you're keeping someone and there's evidence behind it and they're happy for us to [provide mCIMT]* (OT3, organisational culture)
Attention, memory, and decision processes	Thinking ‘mCIMT first’	*What we're kind of getting into, that mindset of ‘oh, we've got a neuro patient with an upper limb issue’, that's the first thing I think about when I go out to see them. Are they appropriate? Do they want to participate? And I talk to the OT, like ‘yes or no?’ And sometimes they'll say no, but anyway that's the first I think about is mCIMT*. (PT2, attention)
Social influences	Importance of expert peer modelling to understand full delivery of mCIMT	*There's a lot of paperwork and parts to it ‐ there was the MAL, and the shaping task, and the homework and the [home skills] assignment‐ I couldn't really get a grasp of what that was until I was there doing it with them. [[REMOVED FOR PEER REVIEW] therapist]*. (PT7, modelling)

Abbreviations: MAL, Motor Activity Log; MC, manager/coordinator; mCIMT, modified constraint‐induced movement therapy; OT, occupational therapist; PT, physiotherapist; RITH, Rehabilitation in the Home; TA, therapy assistant; TDF, Theoretical Domains Framework; WA, Western Australia.

**TABLE 2:** Factors to facilitate sustained provision of mCIMT.

Theoretical domain	Factors to facilitate sustained mCIMT provision	Example quote and associated TDF construct
Theme 1: Clinical practice and knowledge application		
Knowledge	New therapists without knowledge and/or experience of mCIMT require education early into RITH employment	*We have had a couple of OTs who aren't overly aware of the CIMT program and its benefits, … I found myself telling an OT basically how to do it last year*. (TA2, procedural knowledge)
Skills	Embedding regular training and support to account for staff turnover	*I wasn't aware of it today but probably just ongoing training and support and how you embed that because that looks like, to me, a barrier going forward*. (MC1, skills development)
Social/professional role and identity	Therapy assistants support therapist‐led mCIMT programs through increased session frequency and providing feedback on progress	*I think it's that support, so we can get the number of sessions that are required in. I don't see it as us changing, definitely we don't change the shaping task, we don't make them harder, we report back when they're looking easier, … it's very much just facilitating to make sure we're getting the required number of sessions within the week or the fortnight of the task when they're doing it*. (TA3, professional role)
Beliefs about capabilities	Additional training to therapy assistants to build their confidence in explaining mCIMT	*I feel like I've got enough of a basic understanding of it … like I know the purpose of it and I understand what I'm doing with it, but I would really like some more in depth training because I don't have the language to explain it effectively to patients*. (TA4, perceived competence)
Social influences	Greater interdisciplinary communication would increase clarity on program delivery among team members	*There's a couple of times where I thought it would have been useful if we could all get together as a team and discuss how the patient is going, … you just have a quick meeting and everyone gets together and you can hash out anything that is not clear in the notes … I think would definitely help us therapy assistants to understand where everyone's heading as it progresses*. (TA1, social support)
Memory, attention, and decision processes	Using the MAL to support identifying suitable patients	*I've started to do the MAL with a lot more patients than I would or even who are appropriate for this, but I've found that is kind of a nice way in to identifying patients to actually go on a program, if they kind of ‘buy in’ to using the MAL as well*. (OT6, decision making)
Theme 2: Patient challenges and engagement		
Knowledge	Educating stroke survivors and carers about mCIMT prior to discharge home	*If the seed has been planted in hospital before they come to us, the engagement is so much better and the perception of the program is like, ‘oh these guys knew about it! Oh, you know about it! Now you're talking about it! This is really exciting!’ and you get more ‘buy in’. (OT4, knowledge)*
Skills	Ability to ‘sell’ mCIMT in the presence of other rehabilitation goals	*Our physios have come up with the thing of ‘oh the patient only wants to talk to me about walking’, about some sort of little hints and tips to get around that, so you don't only discuss walking with the Physio and upper limb with the OT. Those kinds of things and being able, hints and tips to get carers and families to ‘buy in’ …* (MC6, interpersonal skills)
Environmental context and resources	Enhancing accessibility for stroke survivors with aphasia	*I'm just thinking in terms of a lot of our aphasic patients, the resources that RITH have are like too wordy, they don't make sense, and so a lot of those patients sometimes can get excluded I think from the program, so maybe that would be something we could start to look at in the future to include …* (OT2, resources)
Social influences	Engaging inpatient rehabilitation to develop common language and educate on differing priorities for rehabilitation (i.e., safe for discharge vs. improving UL function)	*Which actually goes against some of the stuff they've done in hospital because they've been taught to try and be independent [using less‐affected arm], and then we're saying ‘we'd rather someone else help than you do it with your other arm’*. (OT3, intergroup conflict)
Beliefs about consequences	Choosing the right time to introduce mCIMT to stroke survivors and families	*Sometimes with our families, they're just so overwhelmed by everything, if we start to ask them to do something as intensive as this, it may not happen, so you've got to choose, or choose the time to introduce …* (PT3, consequents)
Theme 3: Service delivery constraints and suggestions to overcome		
Belief about capabilities	Changing intake procedures and increasing caseloads may influence therapist ability to offer mCIMT	*I think that with the changes with our gatekeeper access into RITH in terms of people referring ‐ when the gatekeeper is gone, potentially more referrals take more time and it takes away from doing really good strong interventions so I guess that is a risk for our stroke cohort, neuro cohort in terms of how much capacity and how much intervention they might get if we end up getting very busy*. (MC3, perceived behaviour control)
Environmental context and resources	Improve resources to be more user‐friendly	*Maybe if we had unlimited amounts of funding and money we could make it a booklet! … so it's not so loose paper everywhere*. (OT2, resources)
Social influences	Leverage therapists with experience and passion in neurological rehabilitation to drive mCIMT provision at their site	*Champions are the ones who have the real* interest … *I think we've got a really good amount of Physios and OTs who come to our program initially because of the neuro experience, and so there could be people who just love that [mCIMT] and want to do that*. (MC3, social support*)*
Emotions	Risk for site champion fatigue	*Even we get champion fatigue, because I'm just thinking we've got champions and champions [for several initiatives]*. (MC4, burn out)
Behavioural regulation	Considering changing strategy due to challenges with staff training and skill development	*we need to approach our delivery differently if it is difficult to capture the part timers, and if you've got the P3s on board, so I was thinking, do you actually have people at each base who are the ones who deliver it and unfortunately those patients have to go there so that is their dedicated role, I don't know if that's feasible …* (MC1, action planning)
Theme 4: Program implementation and integration into routine care		
Skills	Adding mCIMT competency to learning objectives as part of new staff onboarding process	*I think we've been looking at making it an advanced goal for learning objective for our new staff, and for staff that have been re‐credentialed, that maybe it does need to be a learning objective and it might incorporate a training objective, that you have completed a training package or something like that*. (MC4, skill assessment)
Social/professional role and identity	Managers see they have a responsibility to facilitate mCIMT delivery as usual care	*I'd go back to performance development planning, running of meetings I guess, orientation of admission (intake) staff that might be taking referrals to the service, and support for staff I guess to organise their, yeah their training and joint visits and yeah facilitation*. (MC4, leadership)
Environmental context and resources	Ongoing P3 clinician support is critical for therapists delivering their first mCIMT program	*I think keeping the P3 involved even for a one‐on‐one visit, a joint visit with a therapist is probably important in getting it off the ground initially for that therapist, and that patient*. (MC4, resources)
Attention, memory, and decision processes	Flagging suitability for mCIMT at point of referral	*That leads to me saying when you're about to be discharged [from hospital], and you can be identified as being appropriate for CIMT, that it's something that they could say at referral, ‘this person is definitely CIMT!’* (TA2, attention)
Behaviour regulation	Improving data collection to understand gaps in service delivery	*I think it's probably about being able to measure how available your staff are to provide it, how skilled they are to provide it, have they been signed off on the competency, and how many of your stroke patients are receiving it that are eligible, and what you measure as a success*. (MC4, self‐monitoring)
Reinforcement	Sharing success stories within and across teams	*For the ones that I've heard of the patients and the benefits that they've got, it's been a very positive experience. So I think that would be very reinforcing in itself but maybe we're not sharing that well enough*. (MC1, rewards/incentives)
Social influences	RITH is the best service to deliver mCIMT	… I don't feel like many other services after in‐patients, like after RITH that could do it. (OT3, group identity)

Abbreviations: MAL, Motor Activity Log; MC, manager/coordinator; mCIMT, modified constraint‐induced movement therapy; OT, occupational therapist; PT, physiotherapist; RITH, Rehabilitation in the Home; TA, therapy assistant; TDF, Theoretical Domains Framework.

## 4 DISCUSSION

In this study, we explored the complex factors influencing the acceptability and sustained provision of mCIMT among clinicians and allied health managers within a large ESD service, which provides rehabilitation to approximately 700 stroke survivors annually. Given that up to one‐third of stroke survivors are eligible (Christie, Rendell, Fearn, et al., 2023), it is important that stroke rehabilitation services strive to sustainably deliver mCIMT. Mapping the qualitative data to the TDF domains provided a point of comparison with prior survey data from our initial behaviour change intervention (Weerakkody, Emmanuel, et al., 2023). This approach enabled us to identify whether new barriers and enablers had emerged as therapists' knowledge, skills, and confidence improved following the implementation of mCIMT in the service. Our study is unique in its investigation of the perceptions and experiences of clinicians who have been trained and supported to deliver a mCIMT program specific to their ESD rehabilitation context, providing additional insights into the implementation of this highly effective, yet under‐utilised, intervention.

The behaviour change intervention (Weerakkody, Emmanuel, et al., 2023), clinical support, and practice opportunities improved mCIMT delivery by enhancing procedural knowledge and reinforcing skills and confidence among therapists, consistent with prior knowledge translation research (Graham et al., 2006). Clinicians identified other factors influencing their decision to offer mCIMT, such as patient motivation and engagement, and availability of carer support. This finding aligns with previous research, where clinicians who were experienced and confident in delivering mCIMT programs reported patient factors, such as the need to tailor CIMT programs to suit individual needs (Christie et al., 2021). In contrast, clinicians with limited experience primarily report therapist factors rather than patient factors, such as a lack of skills and confidence to deliver mCIMT programs (Fleet et al., 2014). Managers and therapy assistants recognised ongoing barriers for new staff, including lack of knowledge, skills, and confidence, as they were unlikely to have gained experience of mCIMT in other settings. Embedding ongoing and booster training processes are essential to address staff turnover and ensure all therapists, including those with limited mCIMT experience, have opportunities to address these barriers. These strategies are consistent with those employed in other studies implementing complex interventions in rehabilitation services, such as goal setting (Baker et al., 2023) and CIMT (Christie, Rendell, Fearn, et al., 2023), and should be adopted in the RITH service.

Several strategies were described to enhance patient and carer engagement, such as explaining the literature supporting mCIMT to gain ‘buy‐in’, using YouTube clips for education, and promptly adapting programs when challenges arose. However, therapists expressed concern about overwhelming carers by asking them to support mCIMT programs while also navigating the difficult transition home from hospital. This influenced their decision to offer mCIMT despite the stroke survivor being eligible and is consistent with reasons for not offering mCIMT in previous literature (Weerakkody, White, et al., 2023). However, we identified contradictory findings from our qualitative study investigating stroke survivor and carer experiences of mCIMT in the RITH service (Weerakkody et al., 2024), conducted around the same time as this study. Carers did report stress related to caring for a stroke survivor at home; however, supporting mCIMT programs did not add to their stress, and they were happy that the stroke survivor participated in the program. Several carers reported that their workload reduced as the stroke survivor's UL function improved and they became more independent (Weerakkody et al., 2024). The variance between therapist assumptions and carer experience is an example of therapist gatekeeping, which is likely limiting the full provision of mCIMT. This needs to be addressed in future research.

Therapist time constraints were identified as a barrier in our initial survey data (Weerakkody, Emmanuel, et al., 2023) and are a commonly reported challenge to implementing (m)CIMT (Christie, Rendell, Fearn, et al., 2023; Weerakkody, White, et al., 2023) and several other evidence‐based rehabilitation interventions (Jolliffe et al., 2019). Some therapists cited ongoing time constraints as their main reason for not consistently offering the therapy. Others described the positive impacts of interdisciplinary collaboration in sharing the workload and reducing the frequency that each therapist needed to see a patient, which was particularly valued by part‐time staff. A discrepancy emerged between managers' and therapists' views regarding the feasibility of part‐time staff providing mCIMT. Managers reported that they thought mCIMT was too labour‐intensive for part‐time therapists to deliver within a busy and limited caseload. Contrarily, part‐time therapists found that the collaborative practice championed in mCIMT reduced the workload burden associated with providing intensive UL rehabilitation. They also noted that the standardisation of practice set‐up, instigated as part of the behaviour change intervention, led to efficiencies and time‐savings during therapy sessions. Challenging these discrepant views requires education to be provided to management regarding the feasibility of part‐time staff delivering mCIMT programs.

Involving therapy assistants was seen as another key enabler to the sustainability of mCIMT program delivery in the RITH service. Some mCIMT programs have used physiotherapy and/or occupational therapy students in their service delivery model to reduce labour costs and increase clinical time for patients (Binns & Taylor, 2011). Optimal utilisation of therapy assistants in patient care has been an identified barrier across several Australian settings, which has impacted on clarity about therapy assistants' scope of practice and professional identity (Huglin et al., 2021; King et al., 2022). Therapy assistants supporting mCIMT programs in the RITH service facilitated greater utilisation of this intervention, offering additional benefits as they are already embedded into the RITH team and can work under guidance with minimal supervision.

Therapists and therapy assistants repeatedly described the home environment as the ideal context to be providing mCIMT, citing the rich opportunities available to translate practice from therapy sessions into using the stroke‐affected arm outside of scheduled therapy at home. This was viewed as an enabler for mCIMT to be delivered in the RITH service compared with other settings. One previous study has investigated CIMT implementation in the ESD setting; however, all three stroke survivors in this study received their mCIMT in a centre‐based program that they attended following their discharge from the ESD service (Jarvis, 2016). Thus, the perspective of delivering mCIMT in the home environment in subacute stroke is novel in our study. These findings have shone light on the potential additional benefits of mCIMT delivered in this setting.

Managers in the RITH service supported mCIMT delivery but expressed reservations regarding its long‐term sustainability. The role of the P3 neurological clinicians was highly regarded in supporting patient selection, modelling the program delivery, and problem‐solving when challenges arose. However, inequities in access to P3 clinicians across sites emerged as a potential barrier, especially with anticipated service growth over an already large geographical area. Site champions were suggested to overcome this barrier, a strategy that has strong evidence in supporting the implementation of complex behaviour change initiatives (Christie, Rendell, Fearn, et al., 2023; Santos et al., 2022). Some managers proposed engaging therapists passionate about neurological rehabilitation to lead site‐based training and skill development. This was a strategy employed by Christie and colleagues in their implementation of CIMT across nine rehabilitation teams in Sydney, Australia, with excellent outcomes in sustaining increased delivery to eligible stroke survivors (Christie, Rendell, Fearn, et al., 2023). However, others commented that the therapists who would be appropriate for this role often champion other service improvement initiatives, raising concerns about burnout for these high‐performing staff. Another suggestion was to assign dedicated roles to therapists to deliver mCIMT; however, this was refuted by other managers, as this approach deviated from standard caseload allocation processes that are based on geography and had the potential to increase travel distances for those therapists. This strategy would also create inherent risks related to staff turnover, particularly if mCIMT delivery at each RITH site was reliant on a small number of individuals. It was therefore considered not suitable to explore further by the management group.

Managers recognised an improvement in rates of delivery of mCIMT across the service following the behaviour change intervention. However, they felt that it was still under‐delivered at their sites, for which they lacked data to determine what proportion of eligible stroke survivors were being offered mCIMT. This knowledge could support managers in facilitating practice change among therapists in their teams (Dempsey et al., 2022). Christie and colleagues used audit and feedback to facilitate behaviour change in their Sydney‐based CIMT implementation package (Christie, Rendell, Fearn, et al., 2023). Audits were conducted across nine rehabilitation teams, and uptake data were presented to clinicians quarterly, with therapists reporting that this maintained accountability in supporting practice change (Christie, Rendell, Fearn, et al., 2023). Implementing regular audits and feedback in the RITH service could drive continued practice improvements. Managers also suggested a need to better promote the positive outcomes of the mCIMT program across the RITH service to increase therapist engagement and in the wider rehabilitation community to build RITH's reputation as an exemplar service delivering this intervention. Communities of practice were another strategy used across teams in Sydney, Australia, where engaged clinicians shared knowledge and experiences to improve practice across sites (Christie, Rendell, Fearn, et al., 2023). This strategy may also support sustained delivery of mCIMT in the RITH service, warranting further exploration.

### 4.1 Limitations

The field of implementation science is relatively new, and the use of theory to explore implementation studies is recommended, guidance on framework selection remains limited (Lynch et al., 2018; Morris et al., 2019; Nilsen, 2015). The purpose of this study was to identify which factors need to be addressed to enhance the acceptability of mCIMT and sustain its delivery, not to evaluate the success of its implementation. We acknowledge that there are several behavioural frameworks (Lynch et al., 2018), including the Reach, Effectiveness, Adoption, Implementation, Maintenance (RE‐AIM) framework (Glasgow et al., 1999), that could have supported this analysis and eventual process evaluation. Our decision to use the TDF was to determine if new barriers and enablers emerged after the delivery of a behaviour change intervention and compare findings with a prior survey that also mapped barriers and enablers to the TDF. Our familiarity with the TDF also influenced our decision to use it (Atkins et al., 2017; Weerakkody, Emmanuel, et al., 2023). Although we do not feel that this negatively influenced our analysis and interpretation, we acknowledge framework selection as a potential limitation.

Furthermore, our research question considered determinants at the individual and system levels regarding the acceptability of mCIMT in the RITH service. The concept of acceptability is complex, and several definitions exist that can influence the choice of framework used to investigate acceptability (Sekhon et al., 2017). We recognise that the Theoretical Framework of Acceptability (TFA) is another evidence‐based framework that could have been used to investigate acceptability (Sekhon et al., 2017). However, the TFA focusses on acceptability at the level of an individual's beliefs about the value of an intervention. Although the TDF investigates behaviour related to the intervention at the level of the individual, it also considers contextual factors outside of the intervention (Lynch et al., 2018; Nilsen, 2015). We believe that this provided a robust option for data analysis to answer our research question, justifying our decision to use the TDF.

### 4.2 Conclusion

The delivery of mCIMT remains limited in rehabilitation services despite its strong supporting evidence. Greater efforts should be made to integrate evidence‐based interventions like mCIMT into routine care, addressing the complexities of real‐world clinical settings with input from all stakeholders. Successful implementation and sustained delivery of mCIMT in the RITH service could substantially enhance UL function and reduce the burden of care for potentially hundreds of stroke survivors and their carers (Weerakkody et al., 2024). Thus, there is a potential return on investment with clinical and economic benefits that underscores the value of persevering with mCIMT implementation in the RITH service. Based on the data presented, mCIMT was acceptable to be delivered as usual care for eligible stroke survivors in the RITH service. Therapists and therapy assistants reported positive experiences delivering programs and recognised the unique advantages of mCIMT in the ESD setting. To ensure that this success fosters further progress, ongoing strategies are essential to sustain the momentum of positive change and prevent regression to previous practices. Recommendations include ongoing training and education, further resource adaptation, and earlier engagement of stroke survivors and carers. These insights, combined with data from our qualitative study conducted with stroke survivors and carers (Weerakkody et al., 2024), will inform further adaptations to the mCIMT program. The impact of these adaptations will be evaluated in future research to determine the long‐term acceptability, feasibility, and sustainability of mCIMT implementation in the RITH service.

## AUTHOR CONTRIBUTIONS

Conceptualisation: A. W.; Study design: A. W., B. S., E. G.; Recruitment: A. W.; Data collection: B. S., E. G.; Data analysis: A. W., B. S.; Writing manuscript—first draft: A. W.; Writing manuscript—review/editing: A. W., B. S., E. G.

## CONFLICT OF INTEREST STATEMENT

The authors declare no conflicts of interest.

## Supporting information


**Data S1.** **Therapist Semi‐structured focus group guide**.

**Data S2.** **Manager semi‐structured focus group guide**.

**Data S2.** **Therapy Assistant Semi‐structured focus group guide**.

## Data Availability

The data that support the findings of this study are available on request from the corresponding author. The data are not publicly available due to privacy and/or ethical restrictions.
