# Supplementary material for: Translating acceptability to sustained delivery: Clinician and manager perspectives on implementing modified constraint‐induced movement therapy in an early‐supported discharge rehabilitation service
Source: Aust Occup Ther J. 2024 Oct 7;72(1):e12993. doi: 10.1111/1440-1630.12993 (PMC11650006; doi:10.1111/1440-1630.12993)
Supplement: Supplementary file 1 — Data S1. Therapist Semi‐structured focus group guide. [file AOT-72-0-s001.pdf]

Supplementary file 1: Therapist Semi-structured focus group guide

This guide outlines the topic questions that will be discussed within each focus group for RITH physiotherapists, occupational therapists and therapy assistants.

1. Please begin by briefly:<br>a. Introducing yourself<br>b. Your therapy discipline<br>c. Number of years working in rehabilitation<br>d. Whether you have or haven't used mCIMT with a patient in RITH?<br><br>Please only use your first name to protect your anonymity.
2. Can you tell me about the therapies that you use with patients who have upper limb (UL) impairment?
3. What influences your decision-making about the type of UL therapy you choose?
4. Can you tell me about your understanding about mCIMT? *Prompt for:*<br>*a. literature findings, stroke guidelines recommendations*<br>*b. identification of components*<br>*c. intensity*<br>d. *duration*
5. Can you share your opinions and experiences of the RITH mCIMT training program? Did this change your perceptions of mCIMT? *Prompt for training, resource development, and clinical support.*
6. For those of you who have used mCIMT with patients, can you tell me your reasons for choosing this therapy?
7. Do you see any benefits to using mCIMT with patients over other therapies? Can you discuss further?
8. What are some of the difficulties or challenges you've found when using mCIMT?
9. For those of you who haven't used mCIMT, can you discuss some reasons why?
10. Can you describe how you think patients have/would perceive this therapy?
11. Do you feel RITH should be offering mCIMT to appropriate patients as standard care? Why/why not?
12. What do you see as current barriers to routine use of mCIMT for appropriate patients?
13. What do you see as the main enablers for mCIMT succeeding in RITH?
14. Is there anything else you would like to discuss?
15. Thank you for your time.
