# Supplementary material for: Translating acceptability to sustained delivery: Clinician and manager perspectives on implementing modified constraint‐induced movement therapy in an early‐supported discharge rehabilitation service
Source: Aust Occup Ther J. 2024 Oct 7;72(1):e12993. doi: 10.1111/1440-1630.12993 (PMC11650006; doi:10.1111/1440-1630.12993)
Supplement: Supplementary file 2 — Data S2. Manager semi‐structured focus group guide. [file AOT-72-0-s002.pdf]

Supplementary file 2: Manager semi-structured focus group guide

This guide outlines the topic questions that will be discussed in the RITH Coordinator and Area Manager Focus Group.

Thank you all for agreeing to participate in this focus group. Qualitative data into therapist perceptions of mCIMT within their workplace have identified managers as a perceived barrier OR enabler to implementation of mCIMT. There is currently no published data on management perceptions of this therapy.<br>1. Please begin by briefly introducing yourself, your current role in RITH, and your clinical background. Please only use your first name to protect your anonymity.
2. Can you share your understanding of mCIMT in treating patients with upper limb impairment?
The Stroke Foundation provides a “Strong Recommendation” for eligible stroke survivors to be offered mCIMT as part of their rehabilitation. mCIMT is the only UL intervention to receive this level recommendation.<br>3. Do you feel RITH should be offering mCIMT to eligible patients as standard care? Can you explain why/why not?
4. What understanding do you have of the mCIMT implementation program delivered to RITH therapists?
5. Do you feel that therapists are using this therapy appropriately and sufficiently for eligible patients? Can you describe any examples?
6. Do you think there has been an increase in therapists using mCIMT? If so, what do you think has supported this increase?
7. What challenges do you perceive therapists and patients may encounter in undertaking mCIMT programs?
8. What do you think needs to be done to encourage greater uptake of use by therapists?
9. Do you feel there is a role for management in supporting uptake of mCIMT? If so, what role?
10. Are there instances or circumstances that you feel mCIMT should not be offered in RITH despite a patient being eligible?
11. Is there anything else you would like to talk about regarding mCIMT and RITH that we haven’t covered?
12. Thank you for your time.
