# Supplementary material for: Translating acceptability to sustained delivery: Clinician and manager perspectives on implementing modified constraint‐induced movement therapy in an early‐supported discharge rehabilitation service
Source: Aust Occup Ther J. 2024 Oct 7;72(1):e12993. doi: 10.1111/1440-1630.12993 (PMC11650006; doi:10.1111/1440-1630.12993)
Supplement: Supplementary file 3 — Data S2. Therapy Assistant Semi‐structured focus group guide. [file AOT-72-0-s003.pdf]

Supplementary file 3: Therapy Assistant Semi-structured focus group guide

This guide outlines the topic questions that will be discussed within a focus group with Therapy Assistants.

1. Please begin by briefly:<br>a. Introducing yourself<br>b. Number of years working in rehabilitation/RITH<br>c. Whether you have or haven't used mCIMT with a patient in RITH? And how many mCIMT programs you've been involved in?<br><br>Please only use your first name to protect your anonymity.
2. Can you tell me about the upper limb therapies that you provide to stroke patients as part of a Therapy Assistant program?
3. Can you tell me about your understanding about mCIMT? *Prompt for:*<br>*a. literature findings, stroke guidelines recommendations*<br>*b. identification of components*<br>*c. intensity*<br>d. *duration*
4. Can you share your opinions and experiences of the RITH mCIMT training program? Did this change your perceptions of mCIMT? *Prompt for training, resource development, and clinical support.*
5. Do you see any benefits to using mCIMT with patients over other therapies? Can you discuss further?
6. What are some of the difficulties or challenges you've found when using mCIMT with a patient?
7. Can you describe how you think patients have/would perceive this therapy?
8. Do you feel RITH should be offering mCIMT to appropriate patients as standard care?
9. What do you see as current barriers to routine use of mCIMT for appropriate patients?
10. What do you see as the main enablers for mCIMT succeeding in RITH?
11. What role do you see Therapy Assistants play in facilitating mCIMT in RITH? Do you have any ideas on how we could be using our Therapy Assistants better in this process?
12. Is there anything else you would like to discuss?
13. Thank you for your time.
